# iPSCs: A powerful tool for skeletal muscle tissue engineering

**DOI:** 10.1111/jcmm.14292

**Published:** 2019-04-01

**Authors:** María del Carmen Ortuño‐Costela, Marta García‐López, Victoria Cerrada, María Esther Gallardo

**Affiliations:** ^1^ Departamento de Bioquímica Facultad de Medicina Instituto de Investigaciones Biomédicas “Alberto Sols”, Universidad Autónoma de Madrid, Spain, (UAM‐CSIC) Madrid Spain; ^2^ Instituto de Investigación Sanitaria Hospital 12 de Octubre (i+12) Madrid Spain; ^3^ Grupo de Investigación Traslacional con células iPS Instituto de Investigación Sanitaria Hospital 12 de Octubre (i+12) Madrid Spain; ^4^ Centro de Investigación Biomédica en Red (CIBERER) Madrid Spain

**Keywords:** biomaterials, induced pluripotent stem cells, iPSCs, iPS‐skeletal muscle, personalized medicine, regenerative medicine, scaffold, tissue engineering, volumetric muscle loss

## Abstract

Both volumetric muscle loss (VML) and muscle degenerative diseases lead to an important decrease in skeletal muscle mass, condition that nowadays lacks an optimal treatment. This issue has driven towards an increasing interest in new strategies in tissue engineering, an emerging field that can offer very promising approaches. In addition, the discovery of induced pluripotent stem cells (iPSCs) has completely revolutionized the actual view of personalized medicine, and their utilization in skeletal muscle tissue engineering could, undoubtedly, add myriad benefits. In this review, we want to provide a general vision of the basic aspects to consider when engineering skeletal muscle tissue using iPSCs. Specifically, we will focus on the three main pillars of tissue engineering: the scaffold designing, the selection of the ideal cell source and the addition of factors that can enhance the resemblance with the native tissue.

## 1 INTRODUCTION

### 1.1 Skeletal muscle: A brief overview

The most abundant tissue of the human body is skeletal muscle, constituting 40% of the total weight. It is a highly specialized tissue and participates in many dynamic functions, including force generation, locomotion, posture control, respiration and mastication, as well as other metabolic functions such as storage of substrates for other tissues or heat generation for the maintenance of body temperature.^1^ Therefore, skeletal muscle requires a very organized structure and a complex network of capillaries that support the constant flow of nutrients and metabolites.

**Figure 1:**
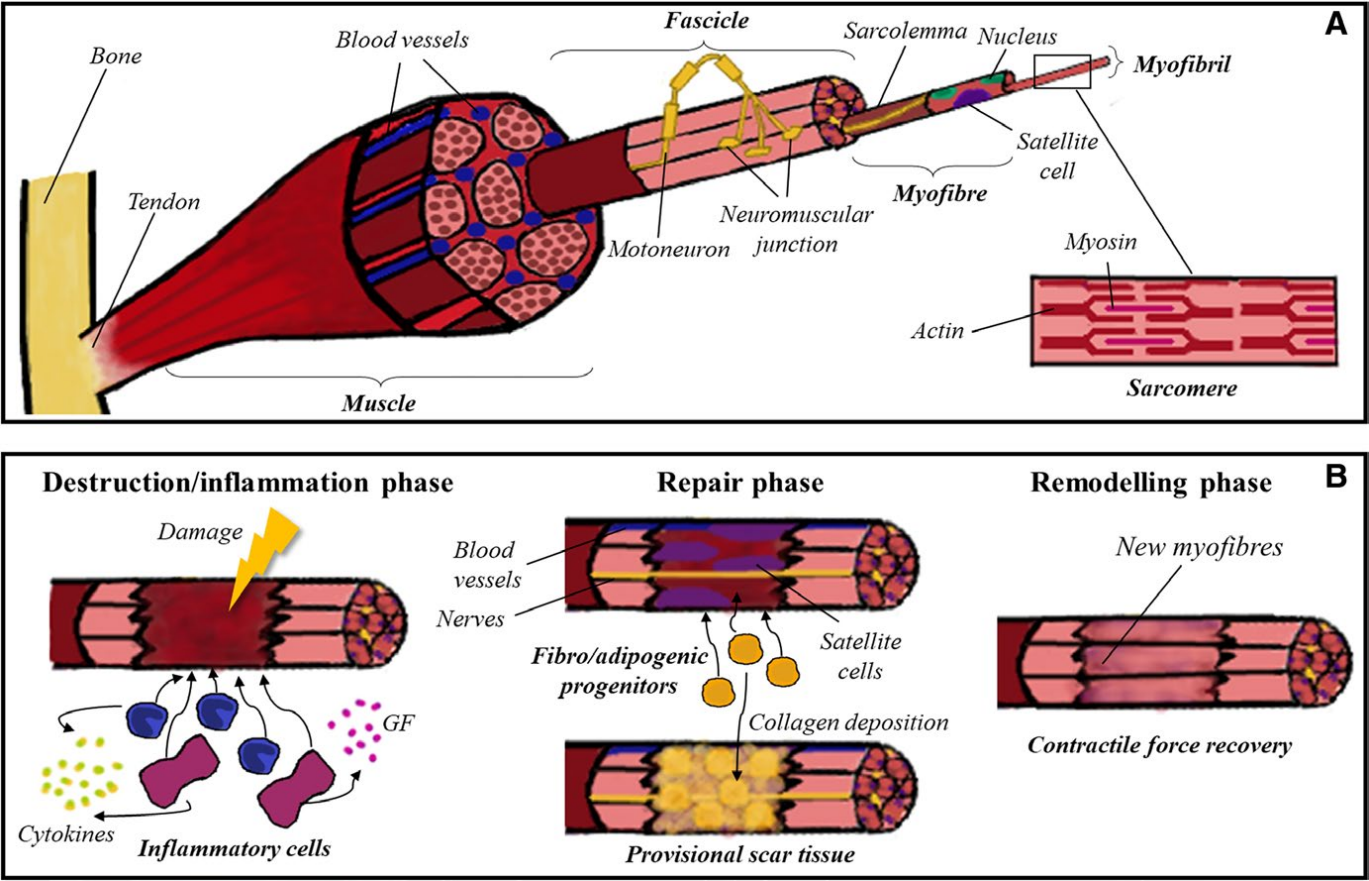
Skeletal muscle tissue. A, Skeletal muscle tissue comprises several bundles of aligned myofibres, each one containing thousands of myofibrils. In the image it is detailed the ultrastructure of the sarcomere, which is the final responsible of the contraction of the muscle due to the movement of myosin and actin myofilaments. B, The repair process of skeletal muscle is divided into three overlapping phases: destruction/inflammation, repair and remodelling. In the first stage, inflammatory cells are recruited to the damaged zone, secreting cytokines and growth factors which attract satellite cells. Then, adjacent blood vessels and nerves invade the healing area, and fibro/adipogenic progenitors co‐operate to form the provisional scar tissue. Finally, new myofibres are formed, and the repairing process concludes when contractile force is recovered

Skeletal muscle is composed of bundles of several aligned multinucleated muscle fibres (also known as myofibres) encoding thousands of myofibrils (Figure 1A).^2^ Each myofibril acts as a contraction unit as a result of the calcium‐dependent movement of thick (myosin) and thin (actin) myofilaments. This structural unit of the myofibril is called sarcomere. A specialized membrane, the sarcolemma, surrounds muscle fibres, where motor neurons carry electrical signals and the flow of calcium ions is regulated. Myofibres are connected to motor neurons forming the neuromuscular junction; this structure includes the pre‐synaptic axon, the synaptic cleft and the post‐synaptic area of myofibres. After the release of acetylcholine in the synaptic cleft, the cell is depolarized and triggers an action potential along the muscle fibre with the release of calcium and the contraction of the muscle.^3^ Vascularization in skeletal muscle is provided by several primary arteries organized in parallel to muscle fibres and numerous arterioles diverging inside them. Myofibres also have a specialized extracellular matrix, composed of basal lamina and reticular lamina.

Satellite cells (so called because of their peripheral location) are a population of muscle stem cells residing between basal lamina and sarcolemma. These cells can either differentiate and proliferate, promoting muscle growth and regeneration or self‐renew to maintain the satellite cells pool. Usually, satellite cells are found in a quiescent state (G_0_ phase) and they activate and expand in response to damage or stress.^4^

### 1.2 The paradigm of tissue regeneration

It is well known that skeletal muscle has a remarkable ability to regenerate after minor injuries such as lacerations, sprains, contusions and small wounds. The repair process comprises three overlapping phases: destruction/inflammation phase, repair phase and remodelling phase (Figure 1B).^5^ After damage, the broken muscle fibres start an inflammatory response with the activation of the complement cascade. Inflammatory cells (neutrophils and macrophages) migrate to the injury zone and start the phagocytosis of necrotic myofibres and cellular debris;^3^ moreover, these cells amplify the inflammatory response releasing cytokines and growth factors and recruiting satellite cells, which differentiate into myoblasts to form new skeletal muscle by fusing to each other or to existing myofibres. In this second stage, adjacent nerves and blood vessels invade the healing area and a population of fibro/adipogenic progenitors start the formation of a provisional scar tissue by collagen deposition.^6^ Finally, new myofibres reorganize themselves and the fibrotic tissue is remodelled to form the definitive muscle structure.

This orchestrated repair concludes successfully with the contractile force recovery. However, this process does not always happen correctly. In cases of volumetric muscle loss (VML) like accidents, surgical procedures and resection of tumours, or even in degenerative processes like muscle dystrophies and sarcopenia, the anatomy of the muscle is so disturbed that the regeneration of the tissue is inefficient, leading to scarring, denervation and even loss of function.

Current treatments for VML, such as muscle flaps or amputation, are limited and show poor efficacy. Nowadays, cell therapy approaches and bioengineering muscle tissues are postulated as great promises for regenerative medicine in the near future.

### 1.3 iPSCs as the key to personalized medicine

The discovery of induced pluripotent stem cells (iPSCs) has marked a milestone in biomedical research. In 2006, murine adult fibroblasts were successfully reprogrammed by introducing only four transcription factors (Oct3/4, Sox2, c‐Myc and Klf4) into the cells using retroviral vectors.^7^ These reprogrammed cells, designated as iPSCs, displayed a typical embryonic stem (ES) cell‐like morphology and growth behaviour, and they exhibited distinctive ES marker genes. Only 1 year later, in 2007, human adult fibroblasts were effectively reprogrammed to become pluripotent using the same four factors.^8^

Mainly due to the integrative nature of retroviral vectors, in the last years there has been an increasing interest in reprogramming by using non‐integrative methods, such as adenovirus, episomal vectors, Sendai virus, synthetic mRNAs and recombinant proteins.^9^ Besides, distinct types of somatic cells like fibroblasts,^10^ urine cells^11^ and even blood cells^12^ have already been reprogrammed with success.

**Figure 2:**
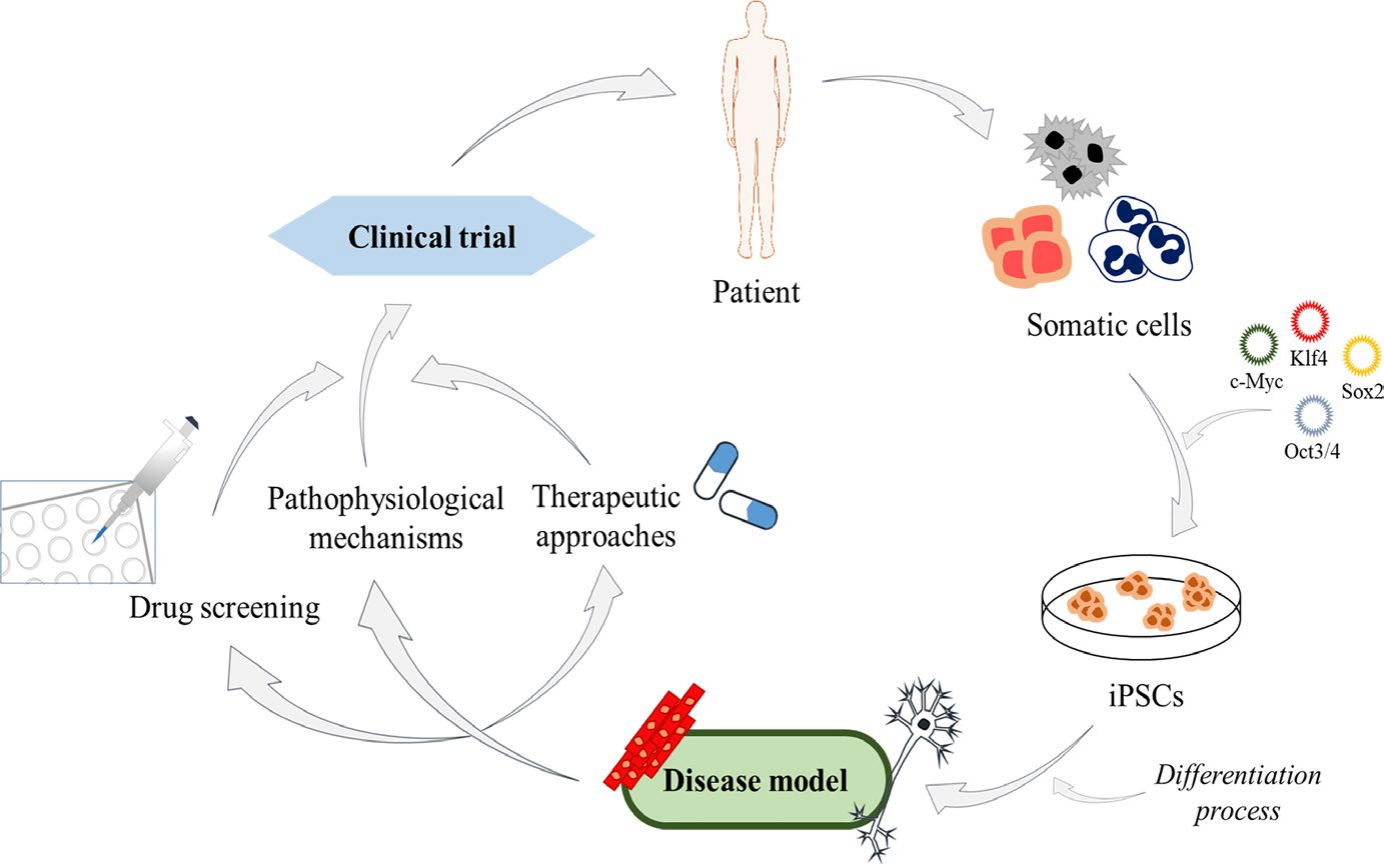
Main applications of patient‐derived iPSCs. Somatic cells from a patient can be reprogrammed using the Yamanaka factors (c‐Myc, Klf4, Sox2 and Oct3/4) into iPSCs. These generated iPSCs can be differentiated into the specific cell type affected in the disease, a step absolutely essential to create a cellular model. The potential applications of those models include drug screening, the elucidation of the pathophysiological mechanisms of the disease and even the discovery of new therapeutic approaches. Finally, all of this can lead to the development of a clinical trial, which could have a very positive impact on the patients themselves

The huge potential of iPSCs is based both on their self‐renewal capacity and their ability to differentiate into practically any cell type. Thus, iPSCs have provided valuable information in the field of stem‐cell differentiation and developmental biology.^13^ However, their contributions go beyond the building up of knowledge. Somatic cells from a patient with a certain disease of interest can be reprogrammed to iPSCs and, afterwards, differentiated into the specific cell type affected, developing a model in vitro that can recapitulate the main features of the disorder. This model could potentially shed light on the pathophysiological mechanisms or possible therapeutic approaches, allowing to perform, for instance, a high‐throughput drug screening, among other applications (Figure 2).^14^, ^15^ Different iPSCs models have already been developed for a wide variety of disorders, like neurological diseases,^16^ optical atrophies,^17^ mitochondriopathies^18^ and even psychiatric disorders.^19^ Nevertheless, complex diseases without a clear genetic background, with low penetrance, late onset or with an unknown affected cellular phenotype are much more complicated to model using this technology.^20^

The differentiation process to the disease target cell type is a pivotal phase in the development of a model. iPSCs have already been differentiated towards an extensive range of cell types, including skeletal muscle cells. Briefly, there are two different approaches to differentiate iPSCs into myogenic precursors.^21^ The first one is based on the overexpression of myogenic transcription factors in the iPSCs, generally MyoD and Pax7, using integrative vectors such as lentivirus.^22^ This kind of approach is highly efficient, but the integration of the vectors can lead to genotoxicity. The other alternative is based on the supplementation with defined factors for a myogenic induction in iPSCs, trying to mimic the embryonic development.^23^ Although it may have a lower efficiency, this approach is safer and allows the use of the differentiated cells for therapeutic applications.

### 1.4 Regenerative medicine and iPSCs: Opening new gateways in cell replacement therapy

As stated before, nowadays there is an increasing necessity for new approaches to deal with VML. Regenerative medicine is highly expected to heal patients with difficult‐to‐treat diseases and physically impaired function, aiming to restore or repair injured or degenerated tissues (and even organs) by the transplantation of cells to the site of injury or degeneration.^24^ To carry out this purpose, it is required to have access to unlimited numbers of functional cells on demand to replenish the cells lost as a result of the disease.^25^

In this sense, human pluripotent stem cells seem to be useful cell sources for cell‐based regeneration, given their capacity to self‐renew and to potentially differentiate into all cell types of adult tissues.^14^ Specifically, iPSCs turn out to be the more suitable alternative for these applications, considering that the cells transplanted into the patients would be directly differentiated from iPSCs generated by reprogramming their somatic cells.

In the context of personalized medicine, iPSCs represent an ideal source to produce patient and disease‐specific adult cells that could be clinically applied in the future. Thus, these iPSCs generated would produce immunologically matched donor cells, which implies the elimination of the immune rejection issue associated with transplant processes.^26^ Moreover, there is a possibility of repairing disease‐causing mutations accurately in iPSCs with the recent advanced tools of genomic edition, being afterwards those iPSCs differentiated and re‐engrafted back into the patient.^27^, ^28^

One of the most remarkable events in regenerative medicine has been the first clinical trial based on human iPSCs, which took place in 2014, to treat age‐related macular degeneration. In this trial, patient iPSCs‐derived retinal pigment epithelium was non‐tumorigenic and the transplant did not cause immune rejection; moreover, data obtained suggest an improvement in visual acuity in the patient.^29^ However, the eye is an immune‐privileged organ, and maybe the translation of these results to other diseases could result in additional and unexpected problems.

A drawback of transplanting cells alone in the site of injury is the fact that many cells are known to be lost in a short time after transplantation, which leads to marginal effects. To overcome this limitation, tissue engineering has emerged as a promising strategy that opens up new therapeutic possibilities.^30^

### 1.5 Tissue engineering: An appealing platform for recreating skeletal muscle

Tissue engineering combines the principles of material sciences, cell transplantation and engineering, aiming the regeneration of failing or damaged tissues.^31^ To accomplish this goal, the idea is to produce suitable biological constructs, preferably autologous, which can be implanted as medical devices inducing the formation of new functional tissues. This entails a promising alternative to traditional surgical procedures for treating VML and its complications.^1^

As it has been mentioned before, the repair of damaged skeletal muscle tissue is limited by the regenerative capacity of the native tissue itself. Tissue engineering represents an appropriate alternative for restoring this tissue, as it is possible to seed and culture cells onto a designed scaffold that mimics the native extracellular matrix, capable of supporting skeletal muscle tissue formation.^32^

There are two main approaches to engineering musculoskeletal tissues: in vitro or in vivo. The in vitro tissue engineering strategy is based on developing muscle constructs in order to transplant the tissue into the patient after differentiation has taken place. On the other hand, the in vivo strategy attempts to inject isolated muscle precursor cells into the damaged tissue site to promote muscle regeneration.^31^, ^33^ Although both techniques have showed to improve muscle function, the in vivo approach requires a large number of cells and additional sites to be injected into.^34^

**Figure 3:**
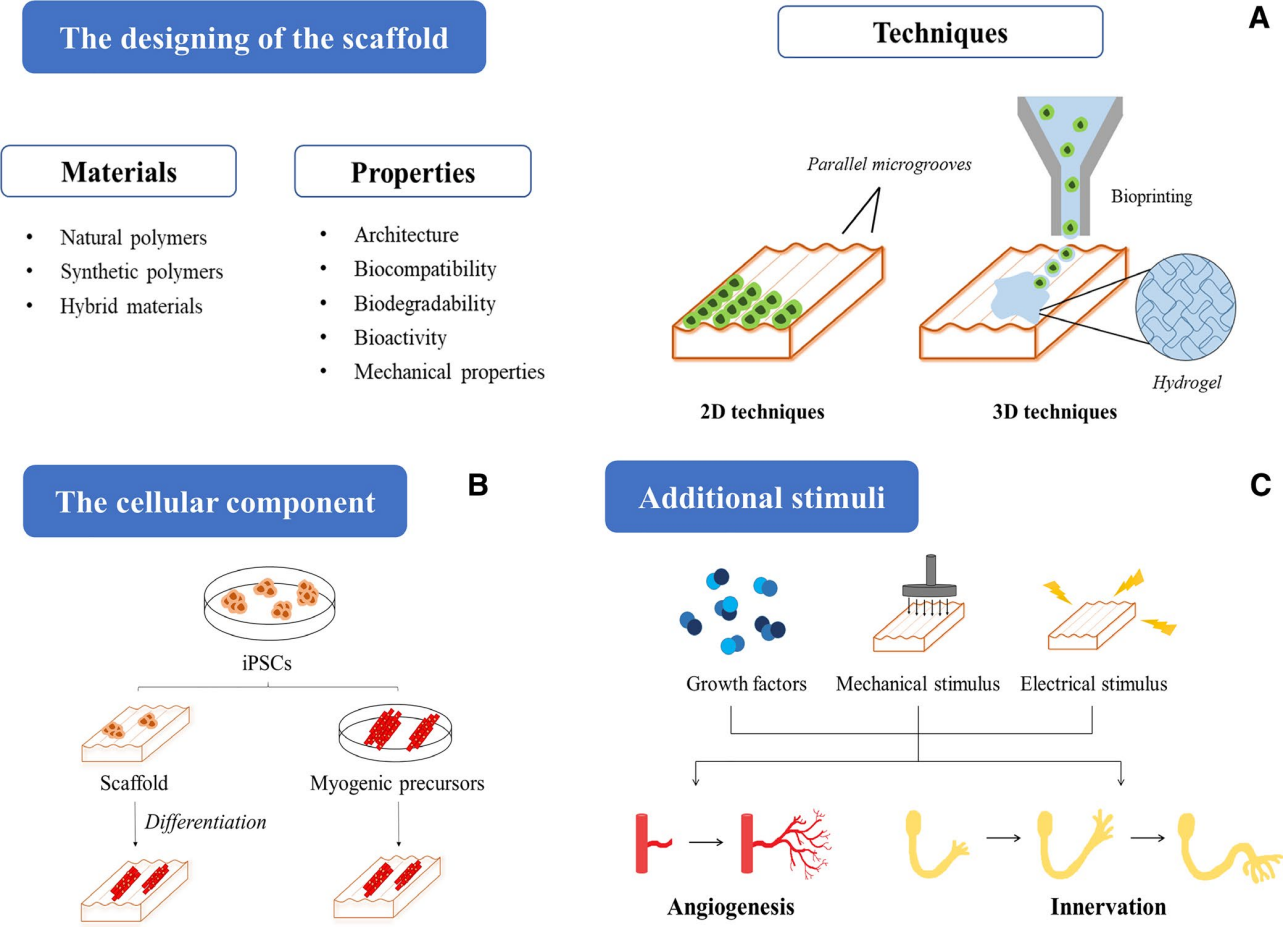
Schematic representation of the three main pillars of skeletal muscle tissue engineering based on iPSCs. A, The scaffolds should present the right properties to ensure the correct engineering of the tissue, and they can be fabricated using natural, synthetic or hybrid polymers. In the image it is highlighted the main two‐dimensional and three‐dimensional techniques that can be employed for the generation of the scaffolds. B, iPSCs can be implemented to this technology following two strategies: either being seeded directly into the scaffold and then differentiated there, or cultivated in the scaffold once differentiated into myogenic precursors. C Finally, with the purpose of achieving vascularization and innervation in the construct, it is possible the addition of bioactive factors, as well as the implementation of mechanical or electrical stimuli

In order to design muscle constructs, it is required to take into account three main pillars: scaffolds, defined cell populations and bioactive agents (growth factors and/or physical stimuli).^35^ In the present review, we will focus on in vitro tissue engineering strategy, delving into the three different aspects on which tissue engineering is supported (Figure 3).

## 2 THE BASICS OF SKELETAL MUSCLE TISSUE ENGINEERING

### 2.1 Designing a suitable scaffold

In order to achieve an engineered muscle tissue similar to the native one, it is essential to design and fabricate an appropriate scaffold. A scaffold is defined as a three‐dimensional (3D) solid biomaterial that plays an indispensable role in tissue regeneration, trying to mimic the extracellular matrix, as both of them present analogous functions.^36^, ^37^ The main features that must be taken into account when designing and choosing a certain scaffold are:

1. Architecture: the scaffold should provide a solid framework, allowing cells to attach, proliferate, migrate and differentiate into myotubes to finally form skeletal muscle constructs.^32^ In this context, apart from the external geometry, it is also essential that the presence of an interconnected pore structure to ensure cell trafficking and an adequate diffusion of nutrients, gases and regulatory factors to the cells; in addition, it is also important to remove waste products out of the scaffold.^38^ Besides, a highly porous structure is also required to have elevated surface density, favouring cell attachment and having enough space to facilitate neovascularization and support new tissue formation.^36^
2. Biocompatibility: the scaffold should be biocompatible, what means that it has to support the appropriate cellular activity being non‐cytotoxic to the cells without inducing any inflammatory responses in the host muscle.^39^ There is a huge number of available surface treatments to optimize the biocompatibility of the scaffold with the tissue, for example using different coatings.^40^ For example, plasma treatment has been used to increase hydrophilicity inducing a better cellularization of the scaffold.^41^
3. Biodegradability: the idea is that the scaffold must remain intact long enough to provide physical support in the early stages of tissue formation, acting as a temporary matrix where the neo‐generated extracellular matrix is going to be deposited. Once cells are organized, the scaffold must biodegrade to gradually be replaced by the newly formed tissue, ideally at a controllable degradation level that approximates the rate of tissue regeneration. It is important that the generated by‐products do not cause any damage to the tissue.^42^ Scaffolds can be modified in order to achieve the desired biodegradability, using techniques as irradiation or oxidation of the material to promote susceptibility to hydrolysis.^43^
4. Bioactivity: the scaffold has to facilitate interactions with cellular components and cell adhesion in order to favour proliferation, migration, differentiation and subsequent tissue organization. These interactions between cells and biomaterials depend on the surface characteristics of the scaffold, such as chemical composition or electric charge.^37^ Scaffolds made from natural biomaterials, as opposed to synthetic ones, are capable of promoting these interactions. Synthetic biomaterial scaffolds need ligands or chemical groups to be incorporated on their surface to enhance cell attachment. Many strategies are feasible for this purpose; for instance, it is possible the addition of extracellular matrix or even collagen coatings.^44^
5. Mechanical properties: the scaffold should provide mechanical integrity and stability to the engineered tissue and similar mechanical properties to those of the tissue that is going to be replaced.^37^ It has to hold enough stiffness to allow cell exposure to relevant mechanical forces, but at the same time appropriate elasticity to accommodate contractile functionality if it is necessary in the tissue.^32^, ^45^

In general terms, an ideal scaffold should fulfil all these criteria mentioned above. In particular, considering that the scaffold will support the engineering of functional skeletal muscle tissue, it should mimic the structure and morphology of the native tissue. As we have previously explained, skeletal muscle tissue is composed of highly oriented and unidirectional aligned myofibres that are disposed forming myotubes, being this organization which dictates muscle tissue function.

The microenvironment, then, is crucial for the maturation, alignment, orientation and definition of the skeletal muscle tissue itself.^46^ Thus, when designing the scaffold it should be taken into account the guidance of cellular orientation to efficiently organize muscle cells and get a functional construct.^47^ Recently, some technologies have been applied to mimic the myotubes alignment: for instance, the fabrication of parallel linear microchannels^48^ or the nano or micro‐patterning of the substrate.^49^, ^50^

Besides, it is of great relevance the advances recently achieved in bioprinting and how this approach has been widely applied to many different tissues. Although there are not many studies in skeletal muscle tissue yet, it would be interesting to implement these procedures, since this approach allows the accurate control of cell distribution at the micrometre scale.^51^

Upon the generation of the muscle construct, two different strategies are feasible with the use of iPSCs. They could be directly seeded onto the scaffold to promote their differentiation, or, conversely, myogenic precursors generated from those iPSCs could be seeded to finish the differentiation process on the 3D environment. One approach or another, a key point to consider is that the scaffold should behave as a muscle stem cell niche, by recreating the native microenvironment in order to promote muscle tissue generation. Therefore, it has to influence myogenic cell differentiation while allowing self‐renewal maintenance of stem cells.^42^ Furthermore, the scaffold should promote angiogenesis, since vascularization is critical for the survival and integration of the engineered skeletal muscle tissue.^31^, ^52^ Finally, the ultimate goal of skeletal muscle tissue engineering is to obtain a biomimetic, functional and contractile system (named myobundle) that mimics the responses of the native tissue as a model.^53^

### 2.2 The art of creating an artificial matrix

Once settled above the main characteristics that a scaffold must fulfil, we are going to focus on the creation of the matrix itself, in particular in the materials and methods used in order to construct the base of the tissue engineered.

The different materials utilized as scaffolds in skeletal muscle tissue engineering can be separated in four main clusters:

1. Natural polymers: they permit cell attachment, interaction and proliferation, and prompt a very limited inflammatory response. However, they have a poor mechanical performance and do not confer an ordered structure.^44^ Some examples widely used as scaffolds are collagen, gelatin, alginate, fibrin and chitosan, each one with their own specific characteristics.
2. Synthetic polymers: one of their main advantages is that their structural and mechanical features can be adjusted to the ones of the tissue engineered. Although they do not count with adhesive sites, they can be modified in order to permit cell adhesion.^54^ Some of the main synthetic polymers employed are poly (glycolic acid) (PGA) and poly (lactic‐co‐glycolic acid) (PLGA).
3. Hybrid materials: the combination of synthetic and natural materials as composites can have many advantages, benefitting from the characteristics of each component. Whereas natural materials tend to contribute to cell adhesion and proliferation, synthetic polymers usually help mimicking the mechanical properties of the tissue engineered.^35^
4. Decellularized scaffolds: they are, basically, tissues or organs that have undergone a process of removal of the cellular and nuclear content in order to leave only the basic structure and composition of the extracellular matrix. Thus, the biomechanical properties of the tissue are conserved.^55^ Apart from being totally biocompatible, one of their main points to highlight is that they count with growth factors and proteins that can be beneficial to the engineering applications, permitting vascularization and angiogenesis. However, a deficient process of decellularization can lead to undesired immune responses.

Apart from the materials, the architecture of the scaffold is another key point to consider. The details to engineer the substrate should be based on the nature of the native tissue itself, and more specifically on the features of the microenvironment and the extracellular matrix that surrounds and supports that tissue. For instance, taking into account the general diameter of the collagen fibres in skeletal muscle tissue, Yang et al. designed the dimensions of the scaffold nanogrooves, determining that 800‐800‐600 nm (ridge width‐groove width‐height) was the optimal ones for the substrate.^56^ The topography of the scaffold is certainly pivotal for the needed uniaxial orientation of the cells, promoting myotube formation, alignment and maturation, and it has been fully proven that flat substrates do not endorse all these features.^56^ Additionally, the elasticity and flexibility of the matrix can be decisive for the maturation of the engineered tissue, permitting contraction and functionality.^57^

The different techniques to obtain this desired anisotropy, both in the micro and nano‐scale, can be differentiated in two groups:

1. Two‐dimensional (2D) techniques: they are based on the modification of the cell‐growing surface itself with parallel microgrooves.^58^ These grooves, which can have different depth and width in order to fulfil the necessities of the tissue engineered, can be induced by distinct methods, like lithography techniques, electrospinning or surface patterning of natural or synthetic polymers. However, cells do not reach a state of complete maturation in this kind of surfaces.
2. 3D techniques: they seek to obtain a geometry closer to skeletal muscle, permitting a further growth and maturation of the cells.^59^ The encapsulation of cells within a hydrogel is one of the most employed techniques. Hydrogels are a family of biocompatible water‐containing materials which tend to form crosslinked networks in 3D.^60^ Although they are highly eligible as scaffolds because of their analogy to biological soft tissues, they do not confer orientation to the cells, and sometimes their density can be a drawback. That is why it is important to take advantage of other adjuvant methods to achieve this alignment. For instance, bioprinting can be extremely useful, permitting to obtain a composite of stem cells and scaffold polymers in hydrogels with the 3D architecture desired.^61^

The generation of 3D devices has the advantage of simulating the environments in which cells grow in vivo, increasing motility and intercellular communication to influence cell fate specification. Furthermore, 3D micropatterned scaffolds can lead to further alignment of myotubes in comparison with that existing in a 2D matrix. Likewise, the phenomenon of angiogenesis requires a 3D microenvironment to achieve the correct scale of complexity of the different blood vessels. Thus, the engineered tissue in a 3D structure can be directly implanted for muscle repair, being tough to achieve with 2D micropatterned surfaces.^62^, ^63^

Apart from all these technologies, the so‐called scaffold‐free approaches are based on the culture of skeletal muscle cells together with satellite cells to obtain monolayers that are able to assembly by themselves in 3D structures.^31^ However, the main limitation of this technique is based on the impossibility to scale the construct.

Although the matrix materials and structure are crucial for the engineering of skeletal muscle, the environment plays a fundamental role as well. Thus, the addition of growth factors and certain peptides in the engineering process can contribute to cell growth, cell differentiation or vascularization, for instance.^40^

### 2.3 The cellular component

Regarding the cell types for being used in skeletal muscle tissue engineering, the preferred ones are autologous cells. The most important features that the cellular component must gather are high proliferative capacity and the ability of efficiently differentiate into skeletal muscle cells, in order to obtain a huge pool of engraftable muscle precursor cells. Satellite cells have been the most regularly studied starting cell reservoir for this application, because of being the native precursors of muscle tissue and having a critical role in physiological processes related to muscle regeneration. However, satellite cells are tedious to isolate and purify, and their proliferative capacity is limited in vitro.^32^, ^64^ Nowadays, most strategies are proposing stem cells for the regeneration of skeletal muscle tissue, for instance mesenchymal stem cells, a multipotent adult stem cell population that has shown capacity for myogenic differentiation.^32^ The problem is that these cells do not present an optimal mode of delivery and their long‐term therapeutic contribution is limited.^65^ In this review, we focus on the use of iPSCs in muscle tissue engineering, as a result of all their advantages, which turn these cells into an improved stem cell source for these therapies.

### 2.4 From iPSCs to skeletal muscle at a glance

Mouse models have been traditionally used to study human diseases. Notwithstanding, they do not always provide the best results. In fact, it is well known that mouse models do not always mimic human disorders in a reliable way. Besides, it is not feasible to perform a high‐throughput drug screening assay as a result of the high number of animals that would be necessary for that purpose. In this sense, iPSCs‐based disease models generated from patients could be a very useful tool to provide personalized treatments and to better understand the genetic and epigenetic features of each patient.^66^ Furthermore, iPSCs derived from healthy donors or edited iPSCs could be an excellent option for cell therapy.^67^

Until date, several attempts of making skeletal muscle in vitro have been performed successfully in both 2D and 3D cultures with skeletal muscle myoblast cell lines, such as the widely used mouse myoblast cell line C2C12,^68^ human skeletal myoblasts^69^ or even with purified satellite cells.^70^

Despite the good results obtained in the aforementioned studies, there are three points that make iPSCs a better choice for engineering skeletal muscle, instead of adult myoblasts: unlimited proliferative capacity, non‐invasive source of donor cells and better results when tested in mouse models.^67^ In the last few years, there have been many attempts to differentiate iPSCs into skeletal muscle precursors, trying to create reliable cellular models of the main muscular diseases. Although overexpression methods such as Pax7 or MyoD transfection lead to higher efficiency rates, genome integration still constitutes the most significant disadvantage for clinic translation. Mondragon‐Gonzalez et al.^71^ generated myogenic progenitors through Pax7 induction which could not only be successfully expanded in vitro, but also achieved myotube maturation. Conversely, small molecule methods do not yield a pure expandable myogenic population, but improved transgene‐free protocols could represent a promising alternative. For instance, van der Wal et al.^72^ propose the combination of a small molecules differentiation followed by fluorescence‐activated cell sorting, in order to achieve expansion of progenitors and finally maturation to myotubes with high fusion index.

In the Table 1 several examples of skeletal muscle differentiation methods from iPSCs have been included. The main achievements in each study, such as gene edition, functionality of the cells/constructs, disease modelling, etc. have also been highlighted.

**Table 1:** Reported examples of skeletal muscle differentiation from iPSCs

Study	Differentiation method	Modelled disease	Some achievements
84	MyoD transfection	Facioscapulohumeral muscular dystrophy	Generation of isogenic control clones. Gene correction (CRISPR/Cas9)
85	MyoD transfection	Duchenne muscular dystrophy	Evaluation of exon skipping in disease‐specific myocytes
72	Small molecules	Pompe disease	Gene correction (CRISPR/Cas9) Large‐scale expansion
71	Pax7 transfection	Myotonic dystrophy 1	NA
86	Small molecules	Duchenne muscular dystrophy	Generation of transplantable myogenic cells
87	MyoD transfection	Muscular dystrophy	High‐throughput drug screening
88	MyoD transfection	Infantile‐onset Pompe disease	NA
89	MyoD transfection	Limb girdle muscular dystrophy type 2C	iPSCs derived from urine cells Gene correction (CRISPR/Cas9)
90	MyoD transfection	Amyotrophic lateral sclerosis	Study of neuromuscular microenvironment and generation of functional myotubes

NA, not applicable.

Until date there are very few examples regarding skeletal muscle tissue engineering using iPSCs. In the first one, authors differentiate iPSCs by transient overexpression of Pax7 and were able to generate functional and contractile myobundles in 3D using a fibrin‐based hydrogel as scaffold.^73^ They achieved Ca^2+^ transients and observed vascularization by the host when grafted these myobundles into a mouse model.

Concurrently, MyoD transfection to differentiate iPSCs from healthy donors and patients with Duchenne muscular dystrophy, limb girdle and congenital muscular dystrophies was performed by Maffioleti et al.^74^ As scaffold, fibrin hydrogels under tension were employed to favour cell alignment. The main achievement of this work has been the co‐culture of skeletal myogenic cells with vascular endothelial cells, pericytes and motor neurons in order to mimic the native tissue, trying to show that the collaboration of these cell types is optimal to obtain a 3D artificial skeletal muscle.

Finally, the ultimate attainment in skeletal muscle tissue engineering has been performed by Osaki et al.^75^ In this group, a 3D amyotrophic lateral sclerosis (ALS) model has been produced by using the organ‐on‐a‐chip technology. For this purpose, a co‐culture of iPSC‐derived motor neurons spheroids, from a patient with sporadic ALS, along with iPSC‐derived three‐dimensional muscle fibre bundles has been performed. This model gets closer to the physiological conditions of the tissue and the pathology of this disease, achieving neuromuscular junction formation, muscle contraction force and synchronized Ca^2+^ transients.

### 2.5 What remains to be done?

Advances in iPSCs technology and tissue engineering have been a breakthrough in the field of regenerative medicine. Many researchers have focused their efforts on the generation of 3D engineered muscle tissues that mimic the native muscle. However, there are still several aspects that need to be improved or explored. One of the main challenges is the proper diffusion of nutrients and oxygen to the entire construct. It has been exhaustively studied that the lack of vascularization limits the size of the tissue; furthermore, an insufficient nutrient supply in the central parts of the structure might cause necrotic cores.^76^ Until date, two different approaches have been performed to improve engineered tissue vascularization: in vivo or in vitro.^77^ In vitro strategies rely on creating constructions capable of making mature vessel networks before implantation, for example co‐culturing fibroblasts, myoblasts and endothelial cells to create a suitable microenvironment to promote vasculogenesis.^78^ Another option could be to incorporate growth factors (such as the vascular endothelial growth factor), into hydrogels or patterned scaffolds to promote angiogenesis. Borselli et al.^79^ devised an injectable and degradable hydrogel, made of alginate and a mixture of VEGF and IGF‐I, and observed that the sustained delivery of these factors enhanced the recovery of ischaemic injured skeletal muscle. In the context of scaffolds, Chiu et al.^52^ proposed a micropatterned polydimethylsiloxane (PDMS) substrate coated with collagen‐chitosan hydrogel containing angiogenic factors as a platform to improve the vascularization of engineered tissues. On the other hand, in vivo strategies consist of generating a viable vessel network after the construct transplantation. At this point it is essential the control of the host response and the regeneration capacity, since host vessels should integrate and replace the engineered construct. Modifying the mechanical characteristics of the scaffold can enhance this balance of the healing phases between the host and the device.^80^

Innervation of the engineered tissue is essential for long‐term survival and for the recovery and maintenance of contractile activity. For this purpose, there are some strategies to enhance force generation and to develop the neuromuscular junctions, for example, co‐culturing myoblasts and neural cells^81^ or using bio‐factors that promote acetylcholine receptors clustering like agrin or laminin.^79^, ^82^

To the best of our knowledge, 3D engineered skeletal muscle must be vascularized and innervated to maturate and resemble native adult muscle function. In addition to these previous strategies, external stimulation techniques such as electrical or mechanical stimulation have been developed. Electrical stimulation can improve myogenic differentiation and maturation, induce myotube contraction or control basic parameters such as cellular morphology, migration or gene expression.^31^ Moreover, electrical stimulation is the key to develop advanced cellular models for drug screening with muscle hypertrophy, properly structural organization, force generation and metabolic flux.^83^ Otherwise, mechanical stimulation can mimic the passive stretch that occurs during embryonic muscle development; furthermore, aged‐related muscle atrophy could be studied by varying tension in mechanical stimulation devices.^59^

Regarding the reproduction of specific muscle diseases, engineered tissue models must involve different types of cells to recapitulate the function and microenvironment of skeletal muscle. This denotes the need for a ‘universal’ culture media that supports the maturation of multiple tissue cells and the stem cell niche requirements. The different cell populations must be pure and should be expandable and the final construct must achieve an adult‐like phenotype.

Once all these challenges are overcome, tissue engineering could be postulated as one of the most promising tools for VML and personalized medicine in muscular diseases.

## 3 CONCLUDING REMARKS

In the last few years there has been an increasing interest in the field of skeletal muscle tissue engineering, turning into one of the most promising tools in regenerative medicine to create functional muscle constructs. This approach opens up a wide range of possibilities: (a) The generation of 3D models to study the physiopathology of certain diseases; (b) The performance of high‐throughput drug screenings and (c) The development of a hopeful option to avoid the problems derived of transplants. Additionally, the combination of all these features with the advantages of iPSCs provides an ideal scenario for the implementation of this technology in personalized medicine.

However, the translation to the clinics is not straightforward, and there are many aspects that must be taken into consideration: (a) iPSCs should be exhaustively verified in order to guarantee that they are not tumorigenic at all. (b) As sometimes iPSCs maintain epigenetic memory from the original somatic cells, it is important to assure that a homogeneous population in terms of epigenetic signatures has been achieved. (c) Immune rejection could be one of the main concerns in the application of iPSCs in the clinic. That is why the creation of a completely characterized iPSCs bank concerning human leukocyte antigens (HLA) could represent in the future a powerful tool to choose the best cell source to create a personalized skeletal muscle construct.

In summary, although the progress in skeletal muscle tissue engineering has been remarkable, there are still many aspects left that could be implemented. For instance, gene editing with CRISPR/Cas9 can be extremely useful to correct genetic defects from patient cells, and subsequently use the generated constructs in transplant procedures. There is no doubt that, once these aspects have been upgraded, skeletal muscle tissue engineering using iPSCs will prompt a huge breakthrough in the field of regenerative and personalized medicine.

## AUTHORS’ CONTRIBUTIONS

M.d.C.‐O.C., M.‐G.L., V.C. and M.E.‐G. contributed in manuscript drafting. M.E.‐G. contributed to manuscript critical revision. All authors have read and approved the final version of the manuscript.

## CONFLICT OF INTEREST

The authors confirm that there is no conflict of interest.
